# Tolerability and safety of the intake of bovine milk oligosaccharides extracted from cheese whey in healthy human adults

**DOI:** 10.1017/jns.2017.2

**Published:** 2017-02-20

**Authors:** Jennifer T. Smilowitz, Danielle G. Lemay, Karen M. Kalanetra, Elizabeth L. Chin, Angela M. Zivkovic, Melissa A. Breck, J. Bruce German, David A. Mills, Carolyn Slupsky, Daniela Barile

**Affiliations:** 1 Department of Food Science and Technology, University of California Davis, Davis, CA 95616, USA; 2 Foods for Health Institute, University of California Davis, Davis, CA 95616, USA; 3 Genome Center, University of California Davis, Davis, CA 95616, USA; 4 Department of Nutrition, University of California Davis, Davis, CA 95616, USA

**Keywords:** Bovine milk oligosaccharides, Gastrointestinal tolerability, Mass spectrometry, Microbiota, Next-generation sequencing, BMO, bovine milk oligosaccharides, GI, gastrointestinal, HMO, human milk oligosaccharides, HNC, Human Nutrition Center, OTU, operational taxonomic unit

## Abstract

Mechanistic research suggests a unique evolutionary relationship between complex milk oligosaccharides and cognate bifidobacteria enriched in breast-fed infants. Bovine milk oligosaccharides (BMO) were recently identified as structurally and functionally similar to human milk oligosaccharides. The present single-blind three-way crossover study is the first to determine the safety and tolerability of BMO consumption by healthy human participants (*n* 12) and its effects on faecal microbiota and microbial metabolism. Participants consumed each supplement (placebo-control; low- and high-BMO doses) for eleven consecutive days, followed by a 2-week washout period prior to initiating the next supplement arm. Low and high BMO doses were consumed as 25 and 35 % of each individual's daily fibre intake, respectively. Safety and tolerability were measured using standardised questionnaires on gut and stomach discomfort and stool consistency. Faecal extracts were profiled for bacterial populations by next-generation sequencing (NGS) and bifidobacteria presence was confirmed using quantitative PCR. Urine was analysed for changes in microbial metabolism using nuclear magnetic resonance spectroscopy (^1^H-NMR). Consumption of both the low and high BMO doses was well tolerated and did not change stool consistency from baseline. Multivariate analysis of the NGS results demonstrated no change in faecal microbiota phyla among the placebo-control and BMO supplement groups. In conclusion, BMO supplementation was well tolerated in healthy adults and has the potential to shift faecal microbiota toward beneficial strains as part of a synbiotic treatment with probiotic cultures that selectively metabolise oligosaccharides.

The intestinal microbiome is recognised as an important determinant of health and has become a critical area of study for functional foods. Probiotics and prebiotics are widely used alternative therapies for intestinal health. Plant-based prebiotics, such as inulin and fructo-oligosaccharides, are commercially available, but their simple structure may lead to non-selective growth of gut microbes. Thus, there is an unmet need for selective, scientifically validated strategies to guide the intestinal microbiome towards protective populations. Mechanistic research has led to the understanding of the unique evolutionary relationship between complex human milk oligosaccharides (HMO) and cognate bifidobacteria enriched in breast-fed infants^(^^1^^)^. HMO are abundant in human milk (1–2 %, w/v), and are structurally diverse. Because humans lack the glycolytic enzymes in the gut to digest HMO, these indigestible carbohydrates reach the colon intact^(^^2^^–^^4^^)^ and function as selective prebiotics for beneficial gut bifidobacteria^(^^5^^–^^8^^)^. The enrichment of bifidobacteria in breast-fed infants is believed to be driven in part through the prebiotic effect of HMO. Interestingly, bovine milk oligosaccharides (BMO), a commercially accessible alternative to HMO, have been recently identified as structurally and functionally similar to HMO^(^^9^^–^^13^^)^, and are preferentially consumed by select bifidobacteria^(^^14^^)^. BMO can be captured from whey permeate as a waste stream from whey protein concentrate production. Capturing the value from this waste stream as a high-value supplement and/or product for both targeted at-risk populations and for the general public could result in significant, and direct economic revenue and health benefits. This study was designed to determine if dairy-derived BMO were safe and well-tolerated when consumed by healthy human participants. The second objective was to determine if two daily doses of BMO enrich faecal bifidobacteria levels. We hypothesised that a diet containing BMO will be well tolerated in a healthy adult population, and will effectively enrich beneficial gut microbes and/or their metabolism. This bacterial enrichment could coincide with markers of BMO metabolism and confer intestinal health benefits in populations with intestinal distress. This pilot study was the first to document the selective stimulation of protective bifidobacteria *in vivo* and is the first step in establishing an intestinal health dossier on the potential efficacy for a food that has substantiated health claims.

## Materials and methods

### Participants

The sample size for this study was based on data by Bouhnik *et al*.^(^^15^^)^ in which intake of both 5 and 10 g/d of short-chain fructo-oligosaccharides by eight different adults over 8 d resulted in significantly higher faecal bifidobacteria by at least 1 log compared with baseline and was well tolerated. Individuals living in the Davis, CA area were recruited to participate in the study and thirty-six individuals were screened for enrolment into the study. Inclusion criteria included: healthy men and women aged 18–40 years, normal fasting glucose and lipid values, fibre intake <30 g/d, faecal bifidobacterial populations <25 % of total faecal bacteria, born by vaginal birth and breastfed for a minimum of 2 months. Exclusion criteria were: regular consumption of high-fibre cereals or fibre supplements, or yogurt containing bifidobacteria (within the last 4 weeks); lactose intolerance or allergies to dairy products or wheat; tobacco use; pregnancy or lactation; presence of gastrointestinal (GI)/malabsorption disorders or autoimmune disease; or use of prescription or over-the-counter medications/supplements that include pre- and probiotics, corticosteroids, anti-obesity agents, laxatives, antibiotics and lipid altering medications. A total of sixteen individuals who met pre-screening criteria visited the UC Davis Ragle Human Nutrition Center (HNC) for a screening visit to provide a fasting blood sample and a stool sample, and to have their height, weight, blood pressure and heart rate measured. Blood samples were analysed for fasting lipid and glucose profiles, and stool samples were analysed for faecal bifidobacteria. Participants also completed an online Muldoon Omega-3 Food Frequency Questionnaire that contains 444 items (Modified Block 2006-Bodnar FFQ, 2006; NutritionQuest/Block Dietary Data Systems) in order to have their average daily fibre intake estimated. Twelve participants eligible for enrolment were instructed to complete a Baecke physical activity questionnaire^(^^1^,^6^^)^ and detailed health history questionnaire. The Baecke physical activity questionnaire was used to account for energy expenditure in estimating the appropriate energy intake level used to calculate the doses of each dietary treatment. The UC Davis Institutional Review Board approved all aspects of the study and written informed consent was obtained from all participants prior to study procedures. This study was registered on clinicaltrials.gov (NCT01814540).

### Study protocol

This was a single-blind, placebo-controlled, crossover trial with three dietary treatment arms administered in the following order for eleven consecutive days each (day 0 to day 10): (1) placebo-control; (2) low-BMO; and (3) high-BMO doses. Each treatment arm was followed by a 2-week washout. The dietary treatments were not randomised in order to eliminate possible carry-over effects by the BMO supplement. Participants arrived after a 10–12 h overnight fast to the HNC on each weekday morning during each supplement arm, filled out questionnaires to determine compliance and GI tolerability and consumed their morning supplement dose with a breakfast *ad libitum* consisting of any of the following: coffee, tea, milk, sugar, juice, fruit, hot or cold cereals; bagels, cream cheese, butter or jam; waffles and syrup; breakfast burritos or breakfast sandwiches. The doses of each treatment were calculated using the Institute of Medicine's recommendation for daily fibre intake (14 g of dietary fibre per 1000 kcal (4184 kJ) consumed)^(^^1^,^7^^)^. Both the placebo-control and low-BMO treatments were calculated as the number of g of carbohydrate equivalent to 25 % of daily fibre intake based on individual energy consumption and the high-BMO dose was calculated as 35 % of daily fibre intake. The placebo-control was composed of Polycose, a glucose polymer powder (Abbott Nutrition) and the pure lactose-free BMO powder was supplied by Hilmar Ingredients. The oligosaccharide composition for the lactose-free BMO supplement powder can be found in Table 1. The glucose polymer and BMO powder were each combined with equal parts of Nesquik chocolate powder and stored in sealable sachets prior to administering to participants. Participants were instructed to consume their daily treatment dose split as two daily doses in the morning with breakfast and before bedtime. Starting on the morning of day 0 (baseline) and on each weekday morning up to and including day 10, participants arrived at the HNC and were administered their half daily dose of the treatment mixed with 120 ml of lactose-free milk by study personnel with breakfast. Participants were provided with lactose-free milk for mixing their evening doses at home. Lactose-free milk was used to reduce any prebiotic effects of lactose in individuals with subclinical symptoms of lactose intolerance and ensure that observed outcomes are solely due to milk oligosaccharides. Participants were instructed to consume both the morning and evening doses at home on weekend study days.

**Table 1.** Oligosaccharide composition of the lactose-free bovine milk oligosaccharide product

Mass [H^+^]	Monosaccharide composition*	Peak area	Abundance (%)
505·1763	3 Hex	22602918·2	19·9
546·2029	2 Hex 1 HexNAc	10677827·66	9·4
634·2189	2 Hex 1 NeuAc	51947266·34	45·7
667·2293	4 Hex	6619221·77	5·8
675·2454	1 Hex 1 HexNAc 1 NeuAc	429503·96	0·4
691·2404	1 Hex 1 HexNAc 1 NeuGc	741547·2	0·7
708·2557	3 Hex 1 HexNAc	4314541·64	3·8
749·2822	2 Hex 2 HexNAc	807377·23	0·7
796·2717	3 Hex 1 NeuAc	1033609·11	0·9
829·2821	5 Hex	4707544·62	4·1
870·3085	4 Hex 1 HexNAc	3588316·79	3·2
911·3352	3 Hex 2 HexNAc	838388·74	0·7
991·335	6 Hex	2944520·4	2·6
1073·388	4 Hex 2 HexNAc	823533·79	0·7
1114·415	3 Hex 3 HexNAc	686701·61	0·6
1153·387	7 Hex	697138·37	0·6
1315·440	8 Hex	264710·79	0·2

* Hex represents glucose or galactose; molecular weight 162·0528. HexNAc represents *N*-acetyl glucosamine or *N*-acetyl galactosamine; molecular weight 203·0794. NeuAc represents sialic acid; molecular weight 291·0954.

### Diet and dietary data

Throughout the entire study period, participants were instructed to refrain from consuming high-fibre diets and fibre supplements and to avoid consumption of yogurt containing *Bifidobacterium*. On the morning of each study day participants filled out a questionnaire regarding their intake of any study-prohibitive foods/supplements/medications within the past 24 h, which was used to determine compliance. To establish a baseline for habitual dietary intake, participants filled out a 3-d diet record to represent two weekdays and one weekend day during the 1-week run-in period prior to initiating each supplement arm. Dietary data were analysed using FoodWorks Nutrient Analysis Software, version 14 (Nutrition Company).

### Gut tolerability and stool consistency

The modified Pedersen GI questionnaire was used throughout the study to estimate GI tolerability, which prompted participants to rate their feelings on gut symptoms including: overall GI discomfort; bothersomeness; rumbling stomach; rumbling gut; belching; nausea; stomach cramps; gut cramps; bloatedness; acid reflux; flatulence; diarrhoea; vomiting; and constipation^(^^1^,^8^^)^. GI symptoms were rated on an interval scale from 0 to 10, with 0 indicating an absence of the symptom, 1–3 as ‘mild’ discomfort; 4–6 as ‘moderate’ discomfort, 7–9 as ‘severe’ discomfort and 10 as ‘extreme’ discomfort. Participants were also prompted to record the duration of the longest-lasting symptom. The GI questionnaire was administered to participants on five occasions on days 0–10 and once on day 11 of each supplement arm. On days 0–10, participants filled out the GI questionnaire after a 10–12 h overnight fast before and 1, 3, 6 and 9 h after consuming the supplement with breakfast. To establish a baseline for GI symptoms, participants filled out the GI questionnaire 1 week before initiating each supplement arm as a run-in period for five consecutive days in the morning before consuming any food or liquids. GI tolerability responses were separated into two groups for analysis purposes: run-in and intervention. Run-in scores were calculated by averaging the responses to each question for the 5 d of questionnaires. Intervention scores were calculated by averaging the responses to each question at 1, 3, 6 and 9 h after the morning treatment for the 11 d of each study arm. To determine if BMO intake altered stool consistency, participants filled out the Bristol Stool Scale^(^^1^,^9^^)^. The Bristol Stool Scale is a seven-point scale that describes hardness to softness of stool with images and worded descriptions. Stool types 1–2 indicate constipation; stool types 3 and 4 are ideal stools (especially type 4), as they are easy to defecate while not containing any excess liquid; and stool types 5, 6 and 7 lean toward diarrhoea. Bowel movements produced were self-recorded, and participants recorded their date, time, and consistency or type. Participants filled out the Bristol Stool Scale daily for 7 d before initiating each study arm and during days 1–10 of each treatment arm. Stool type responses were separated into two groups for analysis purposes: run-in and intervention. Run-in scores were calculated by averaging stool type responses on days −7 to −1 for each treatment arm. Intervention scores were calculated by averaging the responses during the 11 d of each study arm.

### Anthropometric and clinical measurements

Height, weight, blood pressure, heart rate and body temperature were measured at the HNC on day 0 and day 11 of each treatment arm. Height and weight were used to calculate each participant's BMI (kg/m^2^).

### Samples collected

Blood was collected from participants on days 0 and 11 after a 10–12 h overnight fast. Participants were instructed to collect their stool samples at home at three time points: days −2, 4 and 11 of each treatment arm. Since stool samples cannot be collected on demand, participants were instructed to collect the samples within 1–2 d of each time point. Samples were stored in participants’ home freezers until transported on ice packs by the participant or picked up by study personnel. Participants were instructed to collect first morning urine samples at home on days 0, 4 and 11 prior to transport on ice packs to the HNC. Participants were instructed to refrain from consuming any alcohol 24 h prior to stool, urine and blood collections, and to refrain from consuming non-steroidal anti-inflammatory drugs, fish, or oils derived from fish, borage, evening primrose or krill 24 h prior to blood collection. Blood samples were centrifuged for 20 min at 1300 ***g*** and plasma and serum were separated from the erythrocytes. Additional plasma and serum samples were portioned into aliquots and stored at −80°C until analysed. Plasma lipid (TAG; total, LDL- and HDL-cholesterol) and glucose profiles were analysed by the UC Davis Medical Center Pathology Laboratory (Sacramento, CA). Stool samples were stored at −80°C until analysed for total and bifidobacterial measurements. Urine samples were portioned into aliquots and stored at −80°C until analysed for urinary metabolites.

### Compliance

On the morning of each study weekday, participants filled out a questionnaire regarding their intake of any study-prohibitive foods/supplements/medications within the past 24 h and returned their empty evening supplement sachet from the night before. If participants did not consume prohibitive foods/supplements/medications and returned the empty evening dose supplement sachet, they were cleared for compliance and engaged in study activities and received their evening supplement dose for that evening.

### Faecal microbiota

#### Faecal DNA extraction

DNA was extracted from 150 mg of stool sample using the ZR Fecal DNA MiniPrep kit (ZYMO) in accordance with the manufacturer's instructions, which included a bead-beating step using a FastPrep-24 Instrument (MP Biomedicals) for 2 min (23°C) at 6·5 m/s.

#### Next-generation sequencing

DNA samples were prepared as previously described^(^^20^^)^ with the following modifications. Universal barcoded primers with Illumina sequencing adapters (adapters are italicised and the barcode is highlighted in bold) V4F (5′-*AATGATACGGCGACCACCGAGATCTACACTCTTTCCCTACACGACGCTCTTCCGATCT* **ACTGCTGA**GTGTGCCAGCMGCCGCGGTAA-3′) and V4Rev (5′-*CAAGCAGAAGACGGCATACGAGATCGGTCTCGGCATTCCTGCTGAACCGCTCTTCCGATCT*CCGGACTACHVGGGTWTCTAAT-3′) were used to PCR amplify the V4 region of the 16S rRNA gene^(^^20^^)^. PCR reactions were carried out in triplicate and contained 12·5 µl 2x GoTaq Green Master Mix (Promega), 1·0 µl 25 mm-MgCl_2_, 8·5 µl water, 0·5 µl forward and reverse primers (10 µm final concentration) and 2·0 µl genomic DNA. The triplicate reactions were combined and cleaned and DNA concentrations were quantified using the PicoGreen dsDNA Kit. An equimolar composite sample mixture was made, gel purified, and sequenced at the University of California DNA Technologies Core Facility on an Illumina MiSeq sequencing platform.

#### *Bifidobacterium* quantitative PCR

Levels of *Bifidobacterium* were measured by quantitative PCR (qPCR) using the methods of Penders *et al*.^(^^2^,^1^^)^, with primers Bif-F and Bif-R and probe Bif-P and performed on a 7500 Fast Real-Time PCR System (Applied Biosystems). Assays contained 10 µl 2x TaqMan Universal PCR master mix (Applied Biosystems), 1 µl each forward and reverse primers and TaqMan probe, 5 µl water, and 2 µl genomic DNA. All reactions were carried out in triplicate with a non-template control.

#### Sequence analysis

The QIIME software package (version 1.5.0) was used to analyse the results of the Illumina sequencing run. Illumina V4 16S rRNA gene sequences were demultiplexed and quality filtered using the QIIME software package^(^^2^,^2^^)^. Reads were truncated after a maximum number of three consecutive low-quality scores. After quality trimming, reads were removed from analysis if they were <60 bp and the number of ambiguous bases were >3. The minimum number of consecutive high-quality base calls to include a read (per single-end read) as a fraction of the input read length was set at 0·70. Similar sequences were clustered into operational taxonomic units (OTU) with UCLUST software^(^^2^,^3^^)^ and minimum identity of 97 %. The most abundant sequence was chosen to represent each OTU. Taxonomy was assigned to each OTU with the Ribosomal Database Project (RDP) classifier^(^^2^,^4^^)^ with a minimum confidence threshold of 80 % and the RDP taxonomic nomenclature^(^^2^,^5^^)^. OTU representatives were aligned against the Greengenes core set^(^^2^,^6^^)^ with PyNAST software^(^^2^,^7^^)^ with a minimum alignment length of 75 bp and a minimum identity of 75 %.

### Urinary metabolomics

First morning urine samples were analysed by NMR spectroscopy. Urine samples were prepared for NMR analysis by the addition of 65 µl of the internal standard DSS-d6 (2,2,3,3,4,4-d6–3-(trimethylsilyl)-1-propane sulfonic acid) (at 5 mm) to 585 µl urine, and the pH was adjusted to approximately 6·8 by the addition of small amounts of NaOH or HCl. A portion of the sample (600 µl) was placed into a 5 mm NMR tube as described in Slupsky *et al*.^(^^2^,^8^^,^^2^,^9^^)^. Samples were run on a Bruker 600 MHz NMR spectrometer using the NOESY pulse sequence with water saturation of 2·5 s during the prescan delay, a mixing time of 100 ms, 12 parts per million sweepwidth, 2·5 s acquisition time, eight dummy scans, and thirty-two transients. All spectra were zero-filled to 128k data points, and a weighted Fourier transform with application of a 0·5 Hz line-broadening function and manual phase and baseline correction. Metabolite profiles were derived from targeted profiling analysis using NMRSuite v7.6 Profiler (Chenomx, Inc.) as described^(^^30^^)^. All compounds in the database have been verified against known concentrations of reference NMR spectra of the pure compounds and have been shown to be reproducible and accurate^(^^28^^,^^31^^)^.

### Statistics

The change in gut tolerability, stool consistency (intervention – run-in) and anthropometric data (day 11 – day 0), dietary nutrient data collected during the run-in period and bifidobacteria measured by qPCR on day 0, day 4 and day 11 were checked for normality using the SPSS Explore procedure and were transformed appropriately (SPSS v. 22). Normally distributed data for the change in gut tolerability, stool consistency, anthropometric and nutrient variables were analysed by ANOVA. Repeated-measures ANOVA was performed to determine the effect of time, treatment and time × treatment on log-transformed faecal bifidobacteria levels. If repeated-measures ANOVA demonstrated a significant time effect among day 0, day 4 or day 11, a paired-samples two-tailed *t* test was performed to identify the treatment group that reached significance. One-way ANOVA was performed on faecal bifidobacteria at day 0 to determine the presence of a carry-over effect among the three treatment arms. Multiple-comparisons analysis with a Bonferroni adjustment was used to determine differences among the three treatment groups for all ANOVA (*P* < 0·05). To determine differences in bacterial populations among the three treatments, OTU-based and phylogenetic α-diversity metrics were calculated using rarefied datasets. Specifically, the number of sequences in the smallest sample set was drawn randomly ten times from each sample set and the averages reported. Unweighted and weighted UniFrac distances (i.e. phylogenetic β-diversity metrics) were calculated between all pairs of samples. UniFrac-based sample clustering was performed using principal coordinates analysis. To determine which bacterial populations drive separations between the treated and untreated classes, we conducted a linear discriminate analysis (LDA) using the LDA effect size (LEfSe) method^(^^32^^)^. Urine metabolite concentrations were normalised to creatinine and log_10_ transformed prior to analysis. For group analyses, days 4 and 11 of the placebo-control, low-dose and high-dose BMO arms were compared. Multivariate statistical analysis (principal components analysis; PCA) implemented in Simca-P+12 was used to determine if clustering occurred with supplement. Data were mean centred and unit variance scaled. One-way ANOVA with multiple-comparisons analysis with a Bonferroni adjustment was used to confirm significance of clustering in the PCA plot using SPSS v. 22. One-way ANOVA with multiple-comparisons analysis with a Bonferroni adjustment was used to determine the effect of the supplement groups on the percentage change of urinary metabolites ((day 11 – day 0/day 0) × 100 %) (*P* < 0·05).

## Results

### Subjects

A total of twelve participants were enrolled in the trial (*n* 9 males, *n* 3 females). All twelve participants complied and completed the placebo-control arm, ten participants complied and completed the low-BMO arm, and nine participants complied and completed the high-BMO arm. One participant was disqualified from participation in the study in the middle of the second supplement arm for missing several sample collections. A second participant was disqualified from participation in the study in the middle of the second supplement arm after initiating a course of antibiotics to treat streptococcal pharyngitis. A third participant dropped out of the study due to personal commitments after completing the first and second treatment arms. Data are reported for all nine participants who completed all three supplement arms (*n* 6 males, *n* 3 females).

### Dietary treatment doses

During the high-BMO arm, participants consumed significantly higher BMO powder than in the low-BMO arm (mean intake: 13·1 *v*. 9·4 g/d; *P* < 0·0005). Participants in the low-BMO and placebo-control groups consumed similar amounts of BMO and glucose polymer powder, respectively (Table 2).

**Table 2.** Supplement amount (g) per treatment arm (Mean values, standard deviations and ranges; *n* 9 per treatment arm)

Treatment	Mean	sd	Minimum	Maximum
Placebo-control	9·5	1·6	7·0	11·6
Low-BMO	9·4	1·5	6·9	11·5
High-BMO	13·1*	2·2	9·6	16·1

BMO, bovine milk oligosaccharides.

* Mean value was significantly different from those of the placebo-control and low-BMO treatments (*P* < 0·0005).

### Subject characteristics

Participants’ age, change in weight, BMI and blood pressure between each treatment arm were not different (Table 3).

**Table 3.** Baseline participant characteristics prior to each supplemental arm (Mean values, standard deviations and ranges; *n* 9 per treatment arm)

	Placebo-control				Low-BMO				High-BMO			
	Mean	sd	Minimum	Maximum	Mean	sd	Minimum	Maximum	Mean	sd	Minimum	Maximum
Age (years)	23·9	3·2	19·0	30·0	23·9	3·0	19·0	30·0	23·9	3·2	19·0	30·0
Weight (kg)	69·1	14·1	49·9	100·5	69·4	14·0	49·6	99·7	69·7	13·3	50·0	97·4
BMI (kg/m^2^)	22·5	3·3	19·0	30·3	22·6	3·2	19·1	30·0	22·7	2·9	19·3	29·3
Systolic blood pressure (mmHg)	123·6	8·6	110·0	136·0	116·4	11·8	100·0	132·0	118·6	11·8	102·0	141·0
Diastolic blood pressure (mmHg)	77·6	5·3	70·0	87·0	74·1	8·4	61·0	88·0	73·7	4·4	68·0	81·0

BMO, bovine milk oligosaccharides.

### Nutrient intake data

Nutrient intake data estimated from 3-d diet records filled out by participants the week before each treatment arm are presented in Table 4. Of the nutrients estimated, only reported total fat (g/d) and saturated fat (g/d) intake were significantly higher before the placebo-control *v*. the high-BMO arm (*P* < 0·05) (Table 4). Reported energy intake was higher before the placebo-control *v*. high-BMO arm but this difference was not statistically significant (*P* = 0·085).

**Table 4.** Habitual nutrient intake prior to each supplemental arm (Mean values and standard deviations; *n* 9 per treatment arm)

	Placebo-control		Low-BMO		High-BMO	
	Mean	sd	Mean	sd	Mean	sd
Energy						
kcal	2379	568	2094	353	1857	478
kJ	9954	2377	8761	1477	7770	2000
Protein (g)	96·2	21·8	83·9	28·4	77·1	25·3
Protein (% energy)	16·2	1·2	15·8	3·0	16·7	3·9
Carbohydrate (g)	266·7	64·2	243·7	35·4	229·9	71·9
Carbohydrate (% energy)	45·2	6·1	47·4	8·9	49·2	6·5
Dietary fibre (g)	21·5	6·1	22·4	6·5	19·9	5·7
Total sugars (g)	86·6	15·7	97·1	31·8	76·4	34·0
Total fat (g)	84·6^a^	17·6	74·4^a,b^	20·7	60·2^b^	12·4
Fat (% energy)	32·8	6·3	32·1	8·3	30·1	6·4
Saturated fat (g)	29·9^a^	8·0	24·2^a,b^	6·8	21·0^b^	7·6
Saturated fat (% energy)	11·6	2·8	10·4	2·7	10·4	3·3
Monounsaturated fat (g)	26·3	9·7	26·3	11·2	18·7	3·6
Polyunsaturated fat (g)	12·4	6·2	13·8	4·2	9·5	2·0
Cholesterol (mg)	318·0	138·9	225·4	87·5	247·0	115·6
*Trans*-fatty acids (g)	0·6	0·8	0·2	0·2	0·1	0·2
Total *n*-3 fatty acids (g)	1·3	0·7	1·5	0·9	1·0	0·3
Total *n*-6 fatty acids (g)	1·0	1·1	1·7	1·1	1·3	1·2
*n*-6:*n*-3 ratio	0·9	0·9	1·2	0·7	1·3	1·0
Alcohol (g)	21·3	34·5	19·6	19·7	14·4	14·3

BMO, bovine milk oligosaccharides.

^a,b^ Mean values with unlike superscript letters were significantly different (*P* < 0·05).

### Gut tolerability and stool consistency

**Fig. 1.**
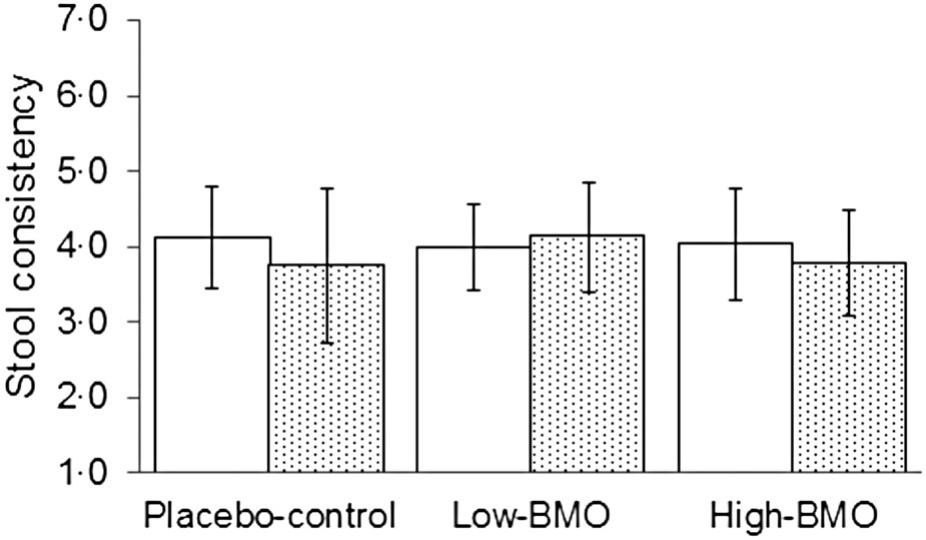
Self-report stool consistency levels by participants during the run-in (□) and intervention (░) period by each study participant. Values are means (*n* 9 per supplemental arm), with standard deviations represented by vertical bars. BMO, bovine milk oligosaccharides.

The mean, standard deviations and ranges for the fourteen GI responses are reported in Tables 5–7. The mean difference between intervention and run-in periods for each of the fourteen symptoms listed in the GI questionnaire were not statistically different (data not shown). Thus, BMO supplementation was equally tolerable compared with the placebo-control. In addition, stool type was not different among the three supplement groups (Fig. 1). The ideal stool consistency (type 4) remained consistent throughout each treatment arm.

**Table 5.** Gut tolerability in response to the placebo-control arm (Mean values, standard deviations and ranges; *n* 9 per treatment arm)

	Run-in				Intervention			
GI symptoms	Mean	sd	Minimum	Maximum	Mean	sd	Minimum	Maximum
Overall GI	0·8	1·0	0·0	2·5	0·3	0·4	0·0	1·2
Bothersome	0·6	0·7	0·0	2·0	0·2	0·2	0·0	0·5
Stomach rumble	0·2	0·7	0·0	2·3	0·1	0·2	0·0	0·6
Gut rumble	0·3	0·7	0·0	2·0	0·1	0·1	0·0	0·2
Belching	0·0	0·1	0·0	0·2	0·1	0·1	0·0	0·2
Nausea	0·0	0·1	0·0	0·3	0·0	0·0	0·0	0·1
Stomach cramps	0·1	0·2	0·0	0·5	0·0	0·0	0·0	0·1
Gut cramps	0·0	0·1	0·0	0·3	0·0	0·1	0·0	0·1
Bloated	0·5	0·7	0·0	2·3	0·1	0·2	0·0	0·4
Acid reflux	0·0	0·0	0·0	0·0	0·0	0·1	0·0	0·2
Flatulence	0·3	0·5	0·0	1·5	0·3	0·4	0·0	1·1
Diarrhoea	0·0	0·0	0·0	0·0	0·0	0·0	0·0	0·1
Vomiting	0·0	0·0	0·0	0·0	0·0	0·0	0·0	0·0
Constipation	0·3	0·6	0·0	2·0	0·0	0·0	0·0	0·1

GI, gastrointestinal.

**Table 6.** Gut tolerability in response to the low-bovine milk oligosaccharide arm (Mean values, standard deviations and ranges; *n* 9 per treatment arm)

	Run-in				Intervention			
GI symptoms	Mean	sd	Minimum	Maximum	Mean	sd	Minimum	Maximum
Overall GI	0·4	0·5	0·0	1·4	0·4	0·4	0·0	1·4
Bothersome	0·2	0·4	0·0	1·4	0·3	0·3	0·0	0·7
Stomach rumble	0·0	0·1	0·0	0·2	0·0	0·0	0·0	0·0
Gut rumble	0·2	0·4	0·0	1·2	0·1	0·1	0·0	0·3
Belching	0·1	0·1	0·0	0·4	0·0	0·1	0·0	0·1
Nausea	0·0	0·0	0·0	0·0	0·0	0·0	0·0	0·0
Stomach cramps	0·1	0·2	0·0	0·6	0·0	0·0	0·0	0·0
Gut cramps	0·1	0·3	0·0	0·8	0·0	0·1	0·0	0·2
Bloated	0·1	0·3	0·0	1·0	0·2	0·2	0·0	0·5
Acid reflux	0·0	0·0	0·0	0·0	0·0	0·0	0·0	0·0
Flatulence	0·3	0·5	0·0	1·4	0·4	0·4	0·0	1·3
Diarrhoea	0·0	0·0	0·0	0·0	0·1	0·1	0·0	0·4
Vomiting	0·0	0·0	0·0	0·0	0·0	0·0	0·0	0·1
Constipation	0·0	0·1	0·0	0·4	0·1	0·2	0·0	0·6

GI, gastrointestinal

**Table 7.** Gut tolerability in response to the high-bovine milk oligosaccharide arm (Mean values, standard deviations and ranges; *n* 9 per treatment arm)

	Run-in				Intervention			
GI symptoms	Mean	sd	Minimum	Maximum	Mean	sd	Minimum	Maximum
Overall GI	0·2	0·4	0·0	1·0	0·4	0·6	0·0	1·8
Bothersome	0·2	0·3	0·0	1·0	0·3	0·3	0·0	0·8
Stomach rumble	0·0	0·0	0·0	0·0	0·0	0·0	0·0	0·1
Gut rumble	0·0	0·1	0·0	0·2	0·0	0·1	0·0	0·3
Belching	0·0	0·0	0·0	0·0	0·0	0·1	0·0	0·2
Nausea	0·0	0·0	0·0	0·0	0·0	0·0	0·0	0·1
Stomach cramps	0·0	0·0	0·0	0·0	0·0	0·0	0·0	0·1
Gut cramps	0·0	0·1	0·0	0·4	0·1	0·1	0·0	0·2
Bloated	0·1	0·2	0·0	0·7	0·2	0·3	0·0	0·8
Acid reflux	0·0	0·0	0·0	0·0	0·0	0·1	0·0	0·2
Flatulence	0·2	0·4	0·0	1·0	0·4	0·4	0·0	1·5
Diarrhoea	0·0	0·0	0·0	0·0	0·0	0·1	0·0	0·3
Vomiting	0·0	0·0	0·0	0·0	0·0	0·0	0·0	0·0
Constipation	0·0	0·0	0·0	0·0	0·1	0·2	0·0	0·5

GI, gastrointestinal

### Faecal microbiota

**Fig. 2.**
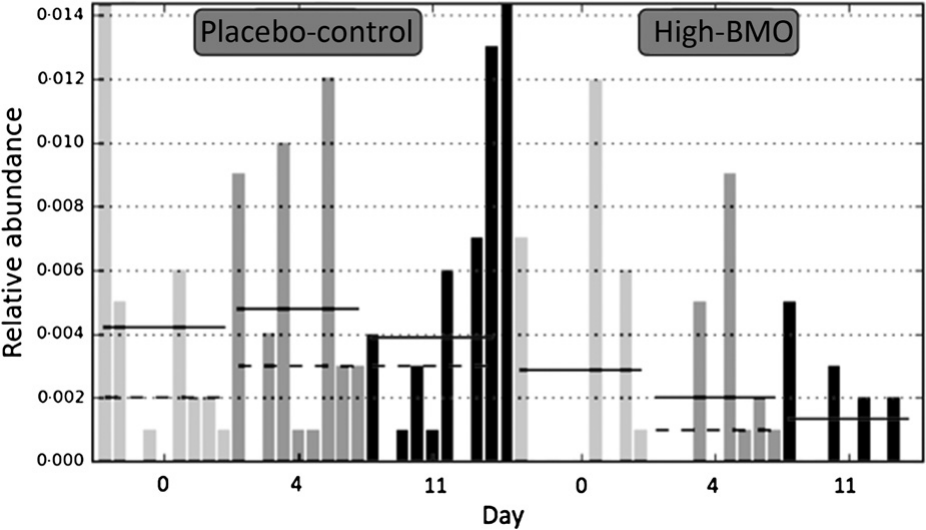
Faecal relative abundance of the family Peptostreptococcaceae in each participant in response to the placebo-control and high-bovine milk oligosaccharide (BMO) supplement arms on days 0 (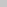), 4 (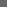) and 11 (■) (*n* 9 per supplemental arm). The linear discriminant analysis effect size between the high-BMO and placebo-control groups for the faecal relative abundance of the family Peptostreptococcaceae was 3·4 (*P* < 0·05).

Next-generation sequencing of 16S rRNA extracted and amplified from subjects’ stool was used to determine faecal microbiota profiles (see Materials and methods). For each of the nine participants who completed the study, the faecal profiles from the placebo-control and high-BMO treatments were compared. LEfSe analysis of the Illumina sequencing data suggests that the microbiota did not change in response to the treatments but the microbiota did cluster according to subject. The lack of a treatment effect was expected after identifying large variation and bacterial diversity in this group of healthy adults. The only taxa that showed changes between treated (high-BMO treatment) and untreated (placebo-control) was Peptostreptococcaceae, with a LEfSe of 3·4 (*P* < 0·05) (Fig. 2).

### Faecal bifidobacteria

**Fig. 3.**
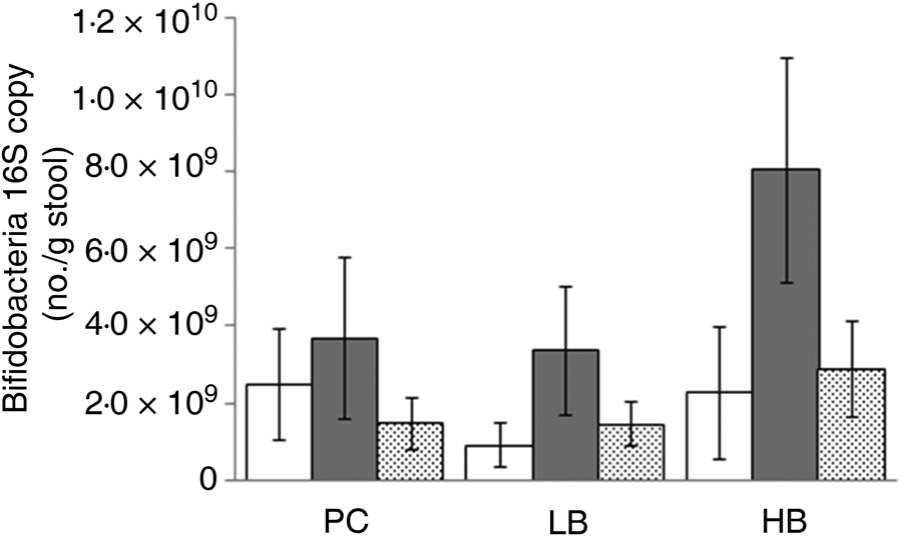
Faecal bifidobacteria levels at day 0 (□), day 4 (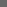) and day 11 (░) in response to the placebo-control (PC), low-bovine milk oligosaccharide (LB) and high-bovine milk oligosaccharide (HB) supplement arms. Values are means (*n* 9 per supplemental arm), with standard deviations represented by vertical bars.

qPCR analysis on DNA isolated from faecal extracts was used to determine if BMO supplementation influenced faecal bifidobacteria levels. Repeated-measures ANOVA identified a significant trend for the effect of time (*P* = 0·06) but not any treatment or time × treatment effects on faecal bifidobacteria levels (Fig. 3). When repeated-measures ANOVA was performed to only compare two time points (day 0 and day 4), the statistical significance for time increased to *P* = 0·01 yet the effect of time disappeared when day 0 and day 11 were compared. Thus the effect of time was observed between day 0 and day 4 but not apparent between day 0 and day 11, suggesting that by day 11, the total level of bifidobacteria returned back to baseline for each of the three treatment arms. A significant difference for faecal bifidobacteria levels between day 0 and day 4 by a paired-samples *t* test could not be determined among any of the treatments. Furthermore, faecal bifidobacteria levels were not different at day 0 for each study arm, suggesting that there was not a carry-over effect by the supplement.

### Urinary metabolomics

**Fig. 4.**
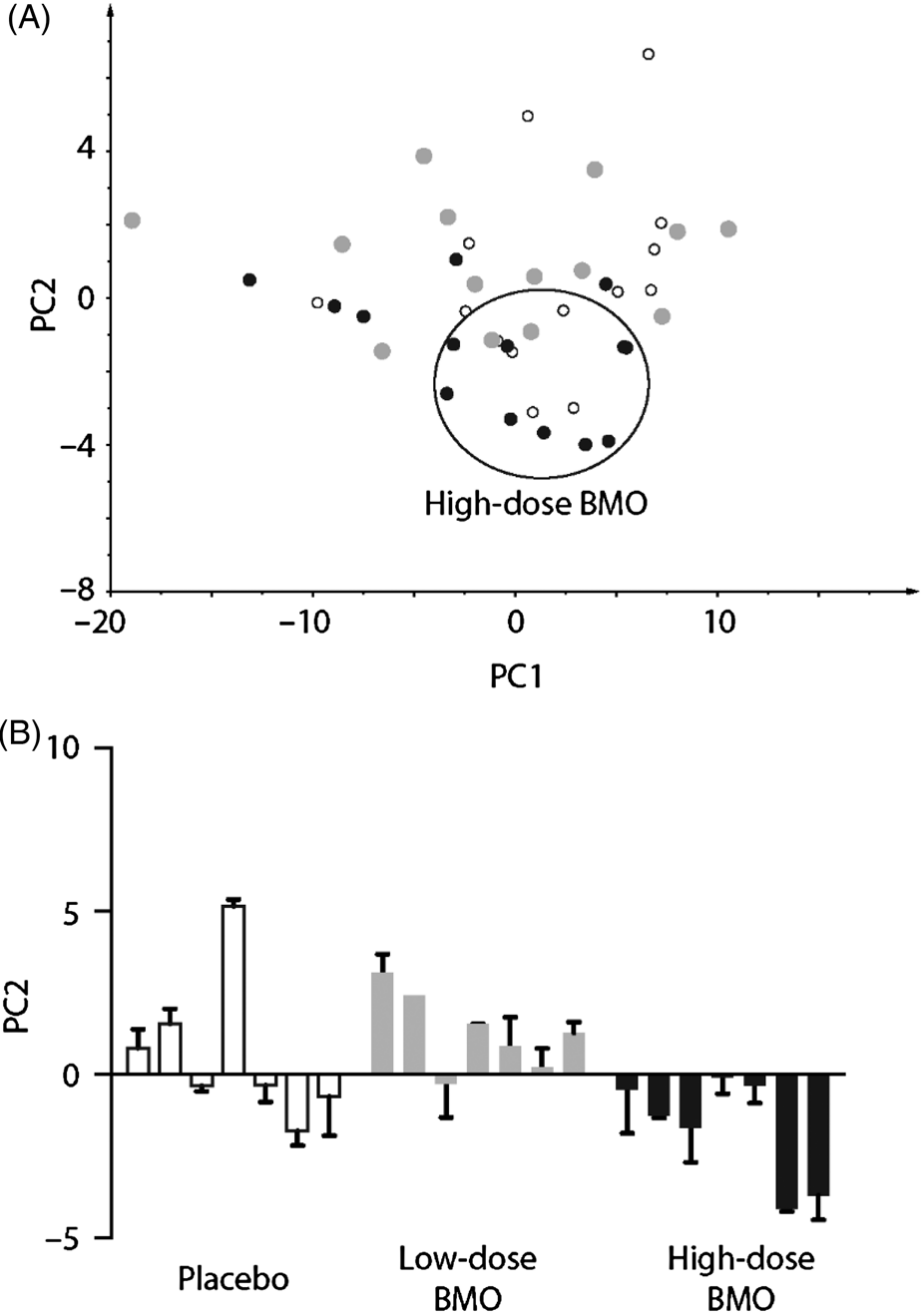
Principal components (PC) analysis of urine metabolite concentrations in different treatment arms. (A) Comparison of days 4 and 11 in the placebo-control (○), low-dose (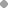), and high-dose (●) bovine milk oligosaccharide (BMO) groups. The variances along PC1 and PC2 were 42 and 5 %, respectively. (B) Mean PC2 value for each of seven subjects who provided a urine sample at all time points during all three treatment arms in the study, with standard errors for days 4 and 11.

**Fig. 5.**
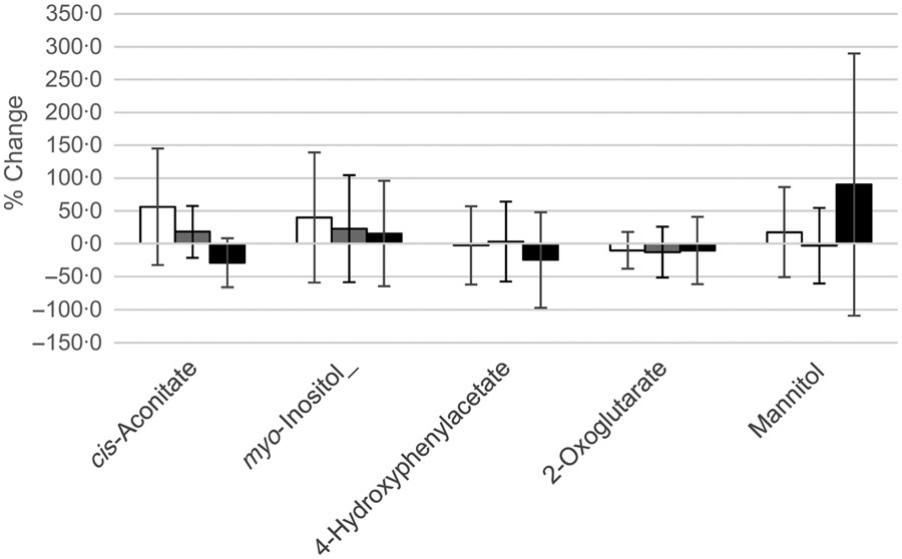
Percentage change between day 11 and day 0 for urinary *cis*-aconitate, *myo*-inositol, 4-hydroxyphenylacetate, 2-oxoglutarate and mannitol across all three supplement arms: placebo-control (□), low-bovine milk oligosaccharide (BMO) (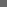) and high-BMO (■). Values are means (*n* 7 per supplemental arm), with standard deviations represented by vertical bars. The mean percentage change for urinary *cis*-aconitate was significantly different in response to the high-BMO compared with the placebo-control (*P* < 0·05).

NMR-based metabolomics was used to assess if changes in urine metabolites could be observed with supplementation. No difference between the low-BMO supplement and the placebo-control could be observed. However, some clustering along principal component 2 (PC2) of the urine after high BMO intake could be observed (Fig. 4(A) and (B)). Analysis of PC2 using repeated-measures ANOVA with Tukey's multiple-comparisons test revealed significance (*P* = 0·003). Pairwise evaluation revealed no difference between placebo-control and the low-BMO group (*P* = 0·7), but significant differences between placebo-control and the high-dose BMO group (adjusted *P* = 0·03), and between the low-BMO and high-BMO groups (adjusted *P* = 0·005). We therefore compared metabolite concentrations between the placebo-control and high-BMO groups for the top variables that were explained by PC2 (4-hydroxyphenylacetate, 1-methylhistidine, *cis*-aconitate, trimethylamine *N*-oxide, 2-oxoglutarate, glutamine, mannitol, and dimethyl sulfone). There were significant differences for urinary *cis*-aconitate and 4-hydroxyphenylacetate at day 11 between the placebo-control and the high-BMO groups (*P* < 0·05) and differences in urinary mannitol at day 11 between the low- and high-BMO groups (*P* < 0·05) (Table 8). When these three urinary metabolites were analysed as a percentage change from baseline, only urinary *cis*-aconitate was significantly reduced in response to the high-BMO compared with the placebo-control (*P* < 0·05) (Fig. 5).

**Table 8.** Urinary metabolites measured at baseline (day 0) and day 11 for each supplement arm (Mean values and standard deviations)

	Placebo-control		Low-BMO		High-BMO	
	Mean	sd	Mean	sd	Mean	sd
*Cis*-aconitate day 0	17·4	5·2	19·2	7·1	20·5	5·1
*Cis*-aconitate day 11	24·2^a^	9·6	20·8^a,b^	4·8	13·8^b^	5·5
*Myo*-inositol day 0	7·6	3·0	6·5	3·5	13·1	4·7
*Myo*-inositol day 11	9·7	6·9	6·8	4·3	12·5	3·3
1-Methylhistidine day 0	162·6	208·1	34·8	44·8	80·0	52·7
1-Methylhistidine day 11	100·7	75·3	84·9	132·4	64·3	63·1
4-Hydroxyphenylacetate day 0	11·1	5·6	9·0	2·5	8·5	4·1
4-Hydroxyphenylacetate day 11	8·9^a^	3·2	8·3^a,b^	4·1	4·4^b^	2·0
2-Oxoglutarate day 0	12·1	5·7	10·2	6·5	8·3	2·9
2-Oxoglutarate day 11	10·7	5·5	7·2	3·7	6·7	3·3
Mannitol day 0	28·2	27·5	11·1	4·4	39·1	45·7
Mannitol day 11	18·6^a,b^	5·5	9·9^a^	6·1	39·1^b^	31·7

BMO, bovine milk oligosaccharides.

^a,b^ Mean values with unlike superscript letters were significantly different (*P* < 0·05).

## Discussion

In this study, we demonstrate for the first time that consumption of whey-derived complex oligosaccharides by healthy participants for eleven consecutive days was safe and well tolerated. During all three treatment arms, GI tolerability symptoms measured throughout each treatment arm did not fluctuate from the run-in period. Additionally, the ideal stool consistency (type 4) remained consistent throughout each study arm. This subject population was screened for healthy GI systems which includes healthy bowel movements. Hence it is not surprising that participants’ stool types did not improve from ideal while consuming the BMO supplement. The low-BMO dose ranged from 6·9 to 11·5 g/d and matched the placebo-control dose that was derived from a readily digestible carbohydrate. The high-BMO dose ranged from 9·6 to 16·1 g/d which is within the dose range used in plant-derived prebiotics. Yet, BMO consumption did not alter GI symptoms from the run-in periods, unlike fructo-oligosaccharides which increased GI intolerance from baseline at doses above 10 g/d^(^^15^^,^^33^^)^. In addition to enhancing GI discomfort, structurally simple prebiotics have been shown to non-selectively enrich other classes of faecal bacteria and not just bifidobacteria^(^^34^^–^^37^^)^.

While there are no commercial sources of these materials today, the existing processing capabilities of cheese and whey manufacturers can be employed to capture BMO from whey permeate and produce sufficient amounts to perform large randomised clinical studies in target populations. BMO supplementation with probiotics was found to be well tolerated and increase intestinal bifidobacteria in infants who were formula fed^(^^38^^)^.

Participants’ metabolic responses did not change over the course of the study period. This is unsurprising due to the short duration of each study arm (11 d) and 2-week washout period. Of the urinary metabolites measured, only *cis*-aconitate changed significantly in response to the high-BMO compared with the placebo-control arm. This could be due to differences in diet that we observed between the placebo-control and the high-dose supplement arm. Indeed, we observed that total saturated fat and total fat were higher in the placebo-control arm *v*. the high-dose BMO arm. Decreases in TCA cycle intermediates such as *cis*-aconitate have been shown to decrease in response to a reduction in fat intake^(^^39^^)^. The reason for not detecting differences in the percentage change of other urinary metabolites is probably due to higher baseline concentrations of these metabolites. Furthermore, the high inter-individual variation for these metabolites may explain why the percentage change for these metabolites was not different among the three treatment arms.

In order to determine if habitual diet during the study period influenced gut tolerability or changes in faecal microbiota, participants filled out 3-d records the week prior to initiating each study arm. Of the dietary variables measured, reported mean dietary total and saturated fat (g/d) were significantly higher during the lead-in period before participants initiated the placebo-control arm compared with the wash-out period before participants initiated the high-BMO supplement arm. Reported mean total energy intake was also higher between these two periods; however, the differences were not statistically significant. There was not a difference in any dietary variable between the period preceding the low-BMO and placebo-control and high-BMO supplemental arms. These observations are unsurprising since the supplemental arms were not randomly assigned and probably due to performance bias that is reduced with randomisation^(^^40^^)^. The reason this study administered the supplemental arms in the following order: placebo-control, low BMO and high BMO was to limit any anticipated carry-over effects by BMO on faecal microbiota or bacterial metabolism. This study is the first to establish that 2 weeks is a sufficient washout period for the intake of BMO.

Ten daily doses of BMO did not substantially change the overall faecal microbial ecology of these healthy adults. This result is not surprising considering that diverse communities found in healthy human adults are stable and resistant to change^(^^41^^)^. Indeed, the lack of substantial change confirms that BMO supplementation is safe and tolerable. The results further suggest that if the intent of BMO supplementation is to change microbial populations, then the BMO will need to be paired with an appropriate probiotic that avidly consumes BMO. It is possible that BMO supplementation alone affects faecal microbial function, rather than overall microbe populations. In a study of supplementation with fermented milk strains, there was no treatment effect on the composition of bacterial species or in the proportional representation of genes encoding known enzymes; however, with RNA sequencing, significant treatment effects were revealed for numerous metabolic pathways^(^^42^^)^. In other words, the bacterial species present did not change in number, but their functions changed significantly. Whether or not BMO supplementation alone affects bacterial function is a question that requires further exploration.
